# Cumulative Impact of Clinical Disease Activity, Biochemical Activity and Psychological Health on the Natural History of Inflammatory Bowel Disease During 8 Years of Longitudinal Follow‐Up

**DOI:** 10.1111/apt.70068

**Published:** 2025-03-09

**Authors:** Christy Riggott, Keeley M. Fairbrass, David J. Gracie, Alexander C. Ford

**Affiliations:** ^1^ Leeds Gastroenterology Institute St. James's University Hospital Leeds UK; ^2^ Leeds Institute of Medical Research at St. James's University of Leeds Leeds UK

**Keywords:** gut‐brain axis, healthcare utilisation, inflammatory bowel disease, prognosis, psychological health, psychology

## Abstract

**Background:**

Common mental disorders, including anxiety and depression, are prevalent in patients with inflammatory bowel disease (IBD) and may be associated with adverse outcomes. However, whether increasing psychological co‐morbidity, in combination with disease activity, exerts a cumulative effect on prognosis is uncertain.

**Aims:**

To assess this in a longitudinal follow‐up study.

**Methods:**

We collected baseline demographic and IBD‐related information, clinical activity using disease activity scores and biochemical activity using calprotectin. Patients were grouped according to the presence or absence of disease activity. Patients in remission or with active disease were subgrouped according to the presence or absence of symptoms of a common mental disorder at baseline. We recorded the occurrence of adverse outcomes over 8.1 years, comparing their occurrence across subgroups using Cox regression.

**Results:**

Among 717 participants with clinical activity data and 187 with clinical and biochemical activity data, rates of adverse outcomes increased with both disease activity and increasing psychological co‐morbidity. Rates of flare or glucocorticosteroid prescription, escalation or death were higher with clinical activity (HR 2.89; 95% CI 1.68–4.93 and 2.52; 95% CI 1.55–4.10 and 6.97; 95% CI 2.43–20.0, respectively) or clinical and biochemical activity (HR 7.26; 95% CI 2.86–18.5, 3.62; 95% CI 1.59–8.25 and 57.3; 95% CI 7.58–433, respectively) and two common mental disorders. Rates of hospitalisation (HR 6.20; 95% CI 1.88–20.4) or hospitalisation and/or intestinal resection (HR 7.46; 95% CI 2.41–23.2) were higher with clinical and biochemical activity and two common mental disorders.

**Conclusion:**

Psychological co‐morbidity and active disease have a cumulative adverse impact on IBD prognosis.

## Graphical abstract

When considered alongside disease activity, psychological co‐morbidity exerts a cumulative adverse impact on IBD prognosis. Strategies to identify and manage co‐existing poor psychological health are therefore necessary to provide holistic care for patients with IBD.

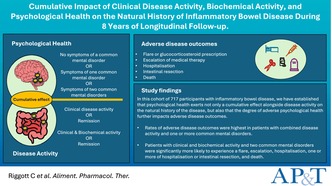

**Abbreviations**
CD: Crohn's disease
CI: confidence interval
FC: faecal calprotectin
HADS: hospital anxiety and depression scale
HBI: Harvey‐Bradshaw index
HR: hazard ratio
IBD: inflammatory bowel disease
PHQ‐15: patient health questionnaire‐15
RCT: randomised controlled trial
SCCAI: simple clinical colitis activity index
UC: ulcerative colitis

## 1 Introduction

Crohn's disease (CD) and ulcerative colitis (UC) are chronic immune‐mediated disorders of the gastrointestinal tract, collectively termed inflammatory bowel disease (IBD). Mucosal inflammation, the hallmark of disease activity, is associated with debilitating symptoms, loss of work productivity and significant morbidity [1, 2]. Prompt recognition and treatment of inflammatory activity is, therefore, the cornerstone of current treatment [3]. The development of advanced medical therapies and cost‐effective non‐invasive biomarkers, such as faecal calprotectin (FC), coincide with a reduction in the frequency of IBD‐related surgeries in Western populations, despite an overall rise in the prevalence of IBD throughout the last two decades [4, 5]. However, although the inflammatory burden is of paramount importance in IBD, substantial healthcare utilisation and high out‐of‐pocket expenses incurred by patients continue to contribute to an increasing socioeconomic burden [6, 7, 8, 9, 10]. In light of the aforementioned developments to improve the management of inflammatory aspects of the disease, this suggests other factors influence the natural history of IBD.

Adverse psychological health influences disease outcomes and healthcare utilisation negatively in the context of chronic illness, including chronic obstructive pulmonary disease, ischaemic heart disease and type 2 diabetes [11, 12, 13]. Similar effects are apparent in IBD, with symptoms of a common mental disorder, such as anxiety or depression, associated with future disease flares, escalation of medical therapy, hospitalisation, IBD‐related surgeries and an attenuated response to biologics [14, 15, 16, 17, 18]. Psychological health also appears to be a driver of increased healthcare utilisation [19, 20, 21, 22, 23], with a Crohn's and Colitis Foundation initiative highlighting that patients with IBD and one or more co‐existing common mental disorders generate double the annual healthcare costs of patients without co‐existing common mental disorders [23]. Of particular relevance, the prevalence of common mental disorders in IBD is significantly higher than in the general population [24]. One‐quarter of patients experience symptoms of depression, and almost one‐third experience symptoms of anxiety, which increase further during periods of disease activity, affecting almost 50% of patients [25].

In ischaemic heart disease, psychological stress influences the development of myocardial ischaemia to a similar magnitude to conventional exercise‐induced stress, with the two exerting a cumulative effect [13]. This suggests psychological health may be of equal importance to physical factors in the context of chronic disease. Given the proportion of patients with IBD exhibiting symptoms of a common mental disorder, these findings bring into question the lack of consensus regarding their identification and appropriate management in routine care [26]. However, a meta‐analysis of over 9000 patients with IBD demonstrated that gut‐brain interactions in IBD are bi‐directional [19]. This highlights an inherent deficit in existing research examining brain‐gut effects; failure to consider co‐existent inflammatory burden, which could be the true driver of adverse disease outcomes in these patients [14, 15, 16, 17].

To date, only one study, to our knowledge, has considered the influence of psychological health in conjunction with inflammatory burden [27]. This reported a cumulative impact of co‐existent poor psychological health and active disease on adverse disease outcomes in IBD, including flare, escalation of medical therapy and mortality. The strength of these associations was similar when disease activity was confirmed biochemically, suggesting genuine braingut effects on the natural history of IBD. A cumulative impact on rarer, but more objective, adverse disease outcomes, including hospitalisation or intestinal resection, was not shown, and a longer duration of follow‐up may be required to assess this. Furthermore, whether increasing psychological co‐morbidity, in combination with disease activity, exerts a cumulative effect on adverse disease outcomes is uncertain. We, therefore, examined this issue in a large cohort of patients with established IBD during an average of 8 years of follow‐up.

## 2 Methods

### 2.1 Participants and Setting

Consecutive patients with an established diagnosis of CD or UC based on endoscopic, histological or radiological findings were invited to participate in a cross‐sectional survey conducted at Leeds Teaching Hospitals NHS Trust between November 2012 and June 2015 [28]. Because participation required completion of a baseline questionnaire, patients who were unable to understand written English were excluded. Due to a lack of reliable scoring tools to assess clinical disease activity among patients with IBD‐unclassified, end ileostomies or colostomies, these individuals were also excluded. Prospective longitudinal follow‐up of all individuals was conducted between September 2014 and October 2023 (REC ref.: 12/YH/0443/AM03). Study findings were reported in line with the STROBE guidelines [29].

### 2.2 Data Collection and Synthesis

The date of study recruitment, type of IBD, and all IBD‐related medications were recorded at baseline in addition to demographic data, including age, sex and lifestyle factors. In addition, we collected data regarding psychological health, including the presence of symptoms of anxiety or depression using the hospital anxiety and depression scale (HADS), with a HADS‐anxiety or HADS‐depression score of ≥ 11 considered abnormal, as suggested by the original validation study [30]. Somatoform symptom reporting was recorded via the patient health questionnaire‐15 (PHQ‐15) [31]. We measured clinical disease activity at baseline using the Harvey‐Bradshaw index (HBI) for CD [32], and the simple clinical colitis activity index (SCCAI) for UC [33]. A score of < 5 was used to define clinical remission in both, as has been suggested previously [32, 34]. All participants were asked to provide a FC sample for analysis (Immundiagnostik, Blensheim, Germany), with biochemical remission defined as a FC of < 100 mcg/g of stool, as supported by international consensus [35], in our main analysis. However, we also used a FC of < 250 mcg/g in a sensitivity analysis.

Participants' medical records were reviewed by two investigators (KMF and CR), both of whom were blinded to baseline questionnaire data to enable objective assessment of disease activity, during longitudinal follow‐up. The following adverse disease outcomes, along with their date of occurrence, were collected: flare of disease activity, based on a physician's global assessment or a prescription for glucocorticosteroids; escalation of medical therapy due to uncontrolled IBD activity; hospitalisation due to uncontrolled IBD activity; intestinal resection due to uncontrolled IBD activity; a composite of either hospitalisation or intestinal resection due to uncontrolled IBD activity (using the first of these events to occur to calculate the time to event); and death. Outcomes occurring within 30 days of recruitment were excluded to minimise any potential association between the presence of a common mental disorder or disease activity at baseline and an impending adverse disease outcome. Alterations made to medical therapy without evidence of uncontrolled IBD activity (e.g., changes made as the result of therapeutic drug monitoring) or surgery for isolated perianal CD were not included as endpoints. Given the duration of follow‐up in the study, it is entirely possible that patients were either lost to follow‐up or moved region and had the care of their IBD taken over by another centre. In these instances, if the event(s) of interest had already occurred, this did not present any issues. However, where the event(s) of interest had not occurred at the point the patient was lost to follow‐up, they were censored, with the event categorised as having not occurred at the last point of follow‐up in our clinic.

### 2.3 Statistical Analysis

Individuals were classified at baseline according to both clinical disease activity and psychological health. Participants were categorised into one of five groups: clinical remission (HBI or SCCAI < 5) without symptoms of a common mental disorder (HADS‐anxiety and HADS‐depression score < 11), clinical remission with symptoms of a common mental disorder (HADS‐anxiety or HADS‐depression score ≥ 11), clinical activity (HBI or SCCAI ≥ 5) without symptoms of a common mental disorder, clinical activity with symptoms of one common mental disorder (HADS‐anxiety or HADS‐depression score ≥ 11) or clinical activity with symptoms of two common mental disorders (HADS‐anxiety and HADS‐depression score ≥ 11). This exercise was then repeated for the subgroup of patients who provided a baseline FC sample to create a further five groups: combined clinical and biochemical remission (FC < 100 mcg/g) without symptoms of a common mental disorder, combined clinical and biochemical remission with symptoms of a common mental disorder, combined clinical and biochemical activity (FC ≥ 100 mcg/g) without symptoms of a common mental disorder, combined clinical and biochemical activity with symptoms of one common mental disorder, and finally combined clinical and biochemical activity with symptoms of two common mental disorders. For these analyses, those with any discrepancy between clinical and biochemical data (e.g., clinical remission but biochemical activity) were excluded from the analysis. We performed a sensitivity analysis with a FC of < 250 mcg/g used to define biochemical remission and ≥ 250 mcg/g used to define biochemical activity.

To assess the impact of baseline clinical or biochemical activity and symptoms of a common mental disorder on disease outcomes, we compared the rates of each of the adverse disease outcomes of interest (flare of disease activity or glucocorticosteroid prescription, escalation of therapy, hospitalisation, intestinal resection, a composite of the two most stringent adverse disease outcomes including only hospitalisation or intestinal resection or death) between the five groups using a *χ* ^2^ test during longitudinal follow‐up. Multivariate Cox regression analysis, controlling for all baseline characteristics including age, sex, marital status, tobacco and alcohol intake, educational level, type of IBD, IBD‐related medications, and level of somatoform symptom reporting according to the PHQ‐15, was performed to identify independent predictors of each of the adverse disease outcomes of interest. A 2‐tailed *p*‐value of < 0.01 was considered statistically significant due to multiple comparisons. Results were expressed as hazard ratios (HR) with a 95% confidence interval (CI). SPSS for Windows version 29.0 (SPSS Inc., Chicago, IL, USA) was used to perform all statistical analyses.

## 3 Results

A total of 760 individuals were recruited, with 717 (94.3%) providing complete clinical activity data at baseline (mean age at baseline 44.0 years, 395 (55.1%) female, 411 (57.3%) CD). A total of 384 (50.5%) of the 760 patients provided a baseline FC measurement, of whom 187 (26.1%) were either in clinical and biochemical remission or had clinical and biochemical activity at baseline (mean age at baseline 49.8 years, 104 (55.6%) female, 96 (51.3%) CD) using an FC of < 100 mcg/g. Using an FC of < 250 mcg/g, 205 (27.0%) patients were either in clinical and biochemical remission or had clinical and biochemical activity at baseline. For the 717 patients providing complete clinical activity data at baseline, the number with longitudinal follow‐up data ranged from 565 (78.8%) for flare of disease activity or need for glucocorticosteroids to 702 (97.9%) for death, with a mean follow‐up in all patients of 8.1 years. Patients with both clinical disease activity and increasing psychological co‐morbidity were significantly less likely to consume alcohol and significantly more likely to report high levels of somatoform symptom reporting (Table 1). The remaining baseline characteristics were similar between groups, including type of IBD, IBD‐related medication use, and location, behaviour or extent of disease. Among 187 patients providing a baseline FC and being in clinical and biochemical remission or having clinical and biochemical activity at baseline, the number with longitudinal follow‐up data ranged from 148 (79.1%) for flare of disease activity or need for glucocorticosteroids to 182 (97.3%) for death, with a mean follow‐up in all patients of 8.3 years. Patients with combined clinical and biochemical activity and increasing psychological co‐morbidity were significantly more likely to smoke tobacco and report high levels of somatoform symptoms (Table S1).

**TABLE 1:** Baseline patient characteristics according to both clinical disease activity and symptoms of a common mental disorder at baseline.

	Clinical remission		Clinical activity			*p* ^a^
	No symptoms of a common mental disorder (*n* = 338)	Symptoms of a common mental disorder (*n* = 85)	No symptoms of a common mental disorder (*n* = 172)	Symptoms of a common mental disorder (*n* = 73)	Symptoms of two common mental disorders (*n* = 49)	
Mean age (SD)	45.6 (18.3)	43.5 (15.1)	43.4 (15.7)	39.6 (13.4)	42.5 (15.7)	0.12
Female sex (%)	166 (49.1)	48 (56.5)	102 (59.3)	47 (64.4)	32 (65.3)	0.029
Married or cohabiting (%)	206 (61.3)	50 (60.2)	109 (64.1)	47 (64.4)	25 (51.0)	0.54
University graduate/professional (%)	107 (32.0)	20 (24.1)	50 (29.2)	15 (20.5)	9 (18.4)	0.11
Tobacco user (%)	49 (14.5)	10 (12.0)	29 (16.9)	18 (25.0)	15 (30.6)	0.013
Alcohol user (%)	234 (69.2)	53 (63.1)	118 (69.0)	43 (59.7)	17 (34.7)	< 0.001
CD (%)	183 (54.1)	50 (58.8)	104 (60.5)	42 (57.5)	32 (65.3)	0.49
CD location (%)						
Ileal (%)	37/183 (20.2)	11/50 (22.0)	20/104 (19.2)	14/42 (33.3)	9/32 (28.1)	
Colonic (%)	61/183 (33.3)	16/50 (32.0)	24/104 (23.1)	7/42 (16.7)	10/32 (31.3)	
Ileocolonic (%)	85/183 (46.4)	23/50 (46.0)	60/104 (57.7)	21/42 (50.0)	13/32 (40.6)	0.21
Non‐stricturing, non‐penetrating CD (%)	157/183 (85.8)	39/50 (78.0)	86/104 (82.7)	31/42 (73.8)	26/32 (81.3)	0.43
Perianal disease (%)	15/183 (8.2)	5/50 (10.0)	14/104 (13.5)	4/42 (9.5)	2/32 (6.3)	0.63
UC extent (%)						
Proctitis	36/155 (23.2)	12/35 (34.3)	14/68 (20.6)	8/31 (25.8)	3/17 (17.6)	
Left‐sided	74/155 (47.7)	13/35 (37.1)	30/68 (44.1)	14/31 (45.2)	10/17 (58.8)	
Extensive	45/155 (29.0)	10/35 (28.6)	24/68 (35.3)	9/31 (29.0)	5/17 (23.5)	0.84
5‐aminosalicylate use (%)	169 (50.0)	39 (45.9)	81 (47.1)	34 (46.6)	18 (36.7)	0.52
Immunomodulator use (%)	121 (35.8)	27 (31.8)	64 (37.2)	28 (38.4)	14 (28.6)	0.73
Biologic use (%)	68 (20.1)	18 (21.2)	30 (17.4)	12 (16.4)	6 (12.2)	0.63
Glucocorticosteroid use (%)	27 (8.0)	9 (10.6)	25 (14.5)	7 (9.6)	9 (18.4)	0.080
High levels of somatoform symptom‐reporting on PHQ‐15 (%)	15 (4.5)	19 (24.7)	35 (21.3)	37 (54.4)	29 (64.4)	< 0.001
FC < 100 μg/g	83/182 (45.6)	16/44 (36.4)	31/77 (40.3)	7/34 (20.6)	12/27 (44.4)	0.092

^a^ One‐way analysis of variance for comparison of continuous data, *χ* ^2^ for comparison of categorical data across all four groups.

### 3.1 Flare of Disease Activity or Need for Glucocorticosteroid Prescription

**FIGURE 1:**
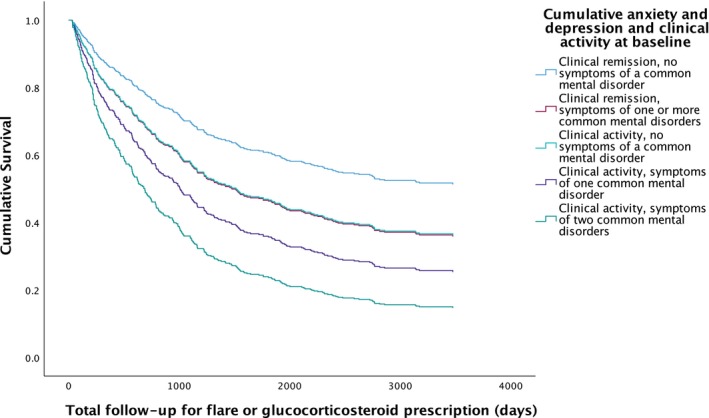
Survival analysis for occurrence of glucocorticosteroid prescription or flare of disease activity according to clinical disease activity status and presence or absence of symptoms of a common mental disorder at baseline.

A total of 306 (54.2%) of 565 patients experienced a flare of disease activity or required a prescription for glucocorticosteroids during a mean follow‐up of 5.0 years in all 565 patients, with a median time to flare or glucocorticosteroid prescription of 1.8 years. Those with clinical activity at baseline and symptoms of one or two common mental disorders at baseline had the highest rates of flare or glucocorticosteroid prescription (60.4% and 78.1%, respectively, *p* = 0.0070 for trend, Table 2). Following multivariate Cox regression, the likelihood of flare or glucocorticosteroid prescription was significantly higher in participants with symptoms of one or two common mental disorders at baseline (HR 2.06; 95% CI 1.30 to 3.26, *p* = 0.0020, and HR 2.89; 95% CI 1.68 to 4.93, *p* < 0.001, Table 2 and Figure 1). Younger age (HR per year 0.98; 95% CI 0.97 to 0.99, *p* < 0.001) was associated with a reduced likelihood, and UC (HR 1.55; 95% CI 1.12 to 2.15, *p* = 0.0080) and glucocorticosteroid use at baseline (HR 1.93; 95% CI 1.26 to 2.96, *p* = 0.0030) an increased likelihood of flare or glucocorticosteroid prescription.

**TABLE 2:** Adverse disease outcomes in patients according to clinical disease activity status and presence or absence of symptoms of a common mental disorder at baseline.

	Clinical remission		Clinical activity			*p*
	No symptoms of a common mental disorder	Symptoms of a common mental disorder	No symptoms of a common mental disorder	Symptoms of a common mental disorder	Symptoms of two common mental disorders	
Flare of disease activity or glucorticosteroid prescription (%)	145/298 (48.7)	45/75 (60.0)	59/110 (53.6)	32/50 (64.0)	25/32 (78.1)	0.0070^a^
Multivariate HR for flare of disease activity or glucorticosteroid prescription (95% CI)	1.00 (reference)	1.54 (1.06–2.23)	1.52 (1.10–2.10)	2.06 (1.30–3.26)^b^	2.89 (1.68–4.93)^b^	< 0.001
Escalation of medical therapy due to uncontrolled IBD activity (%)	157/307 (51.1)	47/78 (60.3)	79/134 (59.0)	32/56 (57.1)	29/38 (76.3)	0.034^a^
Multivariate HR for escalation of medical therapy due to uncontrolled IBD activity (95% CI)	1.00 (reference)	1.43 (1.00–2.05)	1.45 (1.09–1.94)	1.33 (0.86–2.06)	2.52 (1.55–4.10)^b^	0.0020
Hospitalisation due to uncontrolled IBD activity (%)	67/326 (20.6)	26/82 (31.7)	44/168 (26.2)	25/69 (36.2)	17/43 (39.5)	0.0070^a^
Multivariate HR for hospitalisation due to uncontrolled IBD activity (95% CI)	1.00 (reference)	1.45 (0.86–2.42)	1.37 (0.92–2.05)	1.50 (0.88–2.56)	1.53 (0.82–2.83)	0.38
Intestinal resection due to uncontrolled IBD activity (%)	27/326 (8.3)	12/82 (14.6)	24/170 (14.1)	17/71 (23.9)	10/46 (21.7)	0.0020^a^
Multivariate HR for intestinal resection due to uncontrolled IBD activity (95% CI)	1.00 (reference)	1.12 (0.48–2.63)	1.70 (0.94–3.07)	2.41 (1.17–4.97)	1.88 (0.84–4.22)	0.14
Hospitalisation or intestinal resection (%)	67/326 (20.6)	26/82 (31.7)	46/170 (27.1)	26/70 (37.1)	19/44 (43.2)	0.0020^a^
Multivariate HR for hospitalisation or intestinal resection (95% CI)	1.00 (reference)	1.47 (0.88–2.46)	1.40 (0.94–2.09)	1.57 (0.93–2.65)	1.79 (0.99–3.23)	0.22
Death (%)	34/331 (10.3)	6/82 (7.3)	6/170 (3.5)	3/71 (4.2)	8/48 (16.7)	0.011^a^
Multivariate HR for death (95% CI)	1.00 (reference)	1.58 (0.59–4.27)	0.60 (0.23–1.61)	2.11 (0.45–9.77)	6.97 (2.43–20.0)^b^	0.0030

^a^ For comparison across all four groups.

^b^ *p* < 0.001 versus reference category.

**FIGURE 2:**
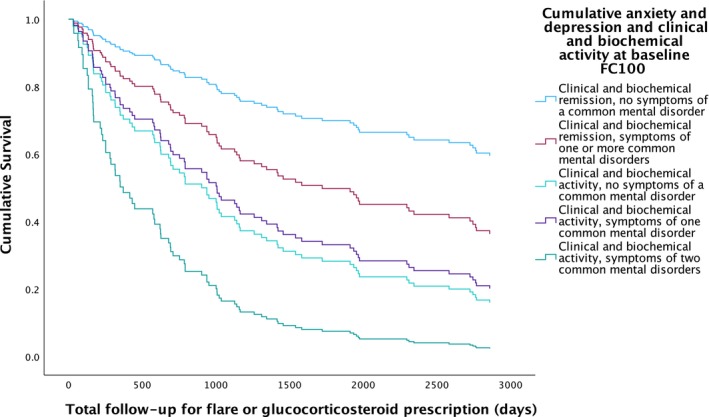
Survival analysis for occurrence of glucocorticosteroid prescription or flare of disease activity according to combined clinical and biochemical disease activity status and presence or absence of symptoms of a common mental disorder at baseline.

When only patients in clinical and biochemical remission or having clinical and biochemical activity at baseline were considered, 84 (56.8%) of 148 patients experienced a flare of disease activity or required a glucocorticosteroid prescription during a mean follow‐up of 5.3 years. Again, patients with clinical and biochemical activity and symptoms of two common mental disorders at baseline had the highest rates of flare or glucocorticosteroid prescription (87.5%, *p* = 0.0080 for trend, Table 3). After multivariate Cox regression, the likelihood of a flare of disease activity or receiving a prescription for glucocorticosteroids remained highest in participants with clinical and biochemical disease activity and symptoms of two common mental disorders at baseline (HR 7.26; 95% CI 2.86 to 18.5, *p* < 0.001, Table 3 and Figure 2), but the rate was also significantly higher in those with clinical and biochemical disease activity and no symptoms of a common mental disorder at baseline (HR 3.54; 95% CI 1.82 to 6.89, *p* < 0.001) and those with clinical and biochemical disease activity and symptoms of one common mental disorder at baseline (HR 3.09; 1.39 to 6.87, *p* < 0.01). Sensitivity analyses with a FC of < 250 mcg/g used to define biochemical remission and ≥ 250 mcg/g to define biochemical activity are provided in Table S2.

**TABLE 3:** Adverse disease outcomes in patients according to combined clinical and biochemical disease activity status, using an FC < 100 mcg/g or ≥ 100 mcg/g, and presence or absence of symptoms of a common mental disorder at baseline.

	Combined clinical and biochemical remission		Combined clinical and biochemical activity			*p*
	No symptoms of a common mental disorder	Symptoms of a common mental disorder	No symptoms of a common mental disorder	Symptoms of a common mental disorder	Symptoms of two common mental disorders	
Flare of disease activity or glucorticosteroid prescription (%)	35/79 (44.3)	11/16 (68.8)	17/24 (70.8)	14/21 (66.7)	7/8 (87.5)	0.019^a^
Multivariate HR for flare of disease activity or glucorticosteroid prescription (95% CI)	1.00 (reference)	1.95 (0.93–4.07)	3.54 (1.82–6.89)^c^	3.09 (1.39–6.87)^b^	7.26 (2.86–18.5)^c^	< 0.001
Escalation of medical therapy due to uncontrolled IBD activity (%)	36/79 (45.6)	10/16 (62.5)	25/31 (80.6)	13/21 (61.9)	8/10 (80.0)	0.0080^a^
Multivariate HR for escalation of medical therapy due to uncontrolled IBD activity (95% CI)	1.00 (reference)	1.51 (0.70–3.24)	3.82 (2.17–6.73)^c^	1.93 (0.88–4.23)	3.62 (1.59–8.25)^c^	< 0.001
Hospitalisation due to uncontrolled IBD activity (%)	11/79 (13.9)	2/16 (12.5)	12/46 (26.1)	8/25 (32.0)	5/13 (38.5)	0.094^a^
Multivariate HR for hospitalisation due to uncontrolled IBD activity (95% CI)	1.00 (reference)	0.88 (0.19–4.18)	1.80 (0.72–4.50)	3.19 (1.12–9.09)	6.20 (1.88–20.4)^b^	0.027
Intestinal resection due to uncontrolled IBD activity (%)	2/79 (2.5)	0/16 (0.0)	6/46 (13.0)	5/25 (20.0)	3/15 (15.0)	0.013^a^
Multivariate HR for intestinal resection due to uncontrolled IBD activity (95% CI)	1.00 (reference)	N/A	4.68 (0.88–24.9)	7.54 (1.14–51.0)	7.18 (0.92–56.2)	0.29
Hospitalisation or intestinal resection (%)	11/79 (13.9)	2/16 (12.5)	12/46 (26.1)	8/25 (32.0)	6/14 (42.9)	0.052^a^
Multivariate HR for hospitalisation or intestinal resection (95% CI)	1.00 (reference)	0.93 (0.20–4.40)	1.86 (0.74–4.65)	3.38 (1.19–9.58)	7.46 (2.41–23.2)^c^	0.0080
Death (%)	11/80 (13.8)	0/16 (0.0)	2/46 (4.3)	2/25 (8.0)	4/15 (26.7)	0.061^a^
Multivariate HR for death (95% CI)	1.00 (reference)	N/A	0.83 (0.16–4.42)	33.6 (3.83–294)^b^	57.3 (7.58–433)^c^	< 0.001

*Note:* N/A; no events, HR not estimable.

^a^ For comparison across all four groups.

^b^ *p* < 0.01 versus reference category.

^c^ *p* < 0.001 versus reference category.

### 3.2 Escalation of Medical Therapy due to Uncontrolled IBD Activity

**FIGURE 3:**
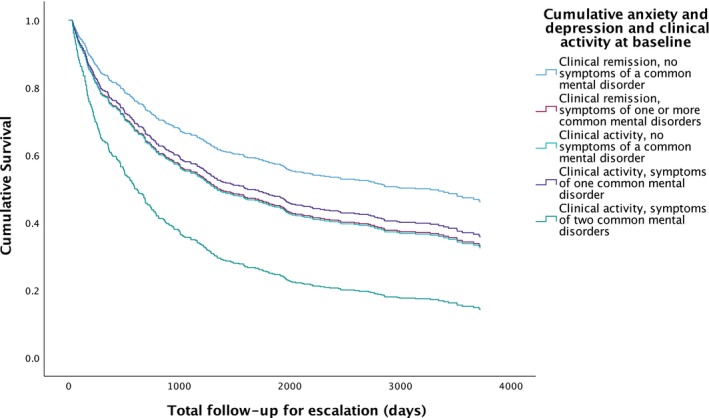
Survival analysis for escalation of medical therapy due to uncontrolled ibd activity according to clinical disease activity status and presence or absence of symptoms of a common mental disorder at baseline.

A total of 344 (56.1%) of 613 patients required escalation of medical therapy due to uncontrolled IBD activity during a mean follow‐up of 4.9 years in all 613 patients, with a median time to escalation of 1.6 years. Rates of escalation were highest (76.3%) in patients with clinical disease activity and symptoms of two common mental disorders at baseline (*p* = 0.034 for trend, Table 2). After multivariate Cox regression, the likelihood of requiring escalation of medical therapy due to uncontrolled IBD activity was significantly higher in patients with clinical disease activity with symptoms of two common mental disorders at baseline (HR 2.52; 95% CI 1.55 to 4.10, *p* < 0.001, Table 2 and Figure 3). Younger age (HR per year 0.98; 95% CI 0.97 to 0.99, *p* < 0.001) was associated with a reduced likelihood of requiring escalation of medical therapy, and baseline glucocorticosteroid use (HR 1.70; 95% CI 1.18 to 2.46, *p* = 0.0040) an increased likelihood.

**FIGURE 4:**
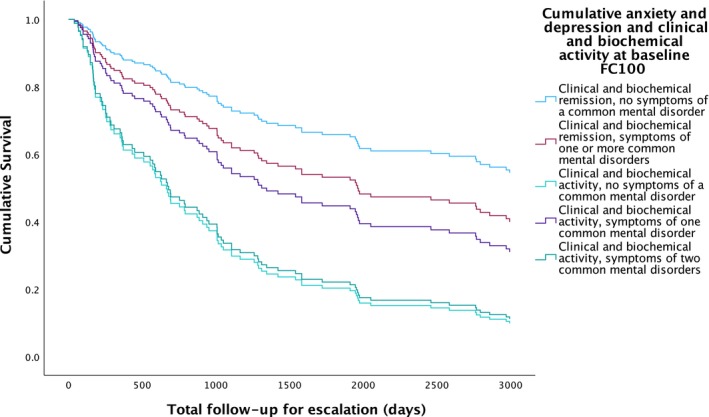
Survival analysis for escalation of medical therapy due to uncontrolled ibd activity according to combined clinical and biochemical disease activity status and presence or absence of symptoms of a common mental disorder at baseline.

When only patients in clinical and biochemical remission or having clinical and biochemical activity at baseline were considered, 92 (58.6%) patients required escalation of medical therapy during a mean follow‐up of 5.1 years in all 157 patients. Rates of escalation due to uncontrolled IBD activity during longitudinal follow‐up were highest in those with clinical and biochemical disease activity and no symptoms of a common mental disorder and those with clinical and biochemical disease activity and symptoms of two common mental disorders (80.6% and 80.0%, respectively, Table 3 and Figure 4). Following multivariate Cox regression, the likelihood of requiring escalation of medical therapy due to uncontrolled IBD activity remained significant in those with clinical and biochemical disease activity at baseline with no symptoms of a common mental disorder (HR 3.82; 95% CI 2.17 to 6.73, *p* < 0.001, Table 3) and those with biochemical disease activity and symptoms of two common mental disorders (HR 3.62; 95% CI 1.59 to 8.25, *p* < 0.001). Sensitivity analyses with a FC of < 250 mcg/g used to define biochemical remission and ≥ 250 mcg/g to define biochemical activity are provided in Table S2.

### 3.3 Hospitalisation due to Uncontrolled IBD Activity

In total, 179 (26.0%) of 688 patients were hospitalised due to uncontrolled IBD activity during a mean follow‐up of 7.0 years in all patients, with a median time to hospitalisation of 2.3 years. Rates of hospitalisation were significantly higher in those with clinical disease activity and symptoms of one or two common mental disorders at baseline (36.2% and 39.5%, respectively, *p* < 0.007 for trend, Table 2). However, the likelihood of requiring hospitalisation was not significantly higher in any of the groups after multivariate Cox regression, although it remained highest in those with clinical disease activity and symptoms of one or two common mental disorders at baseline (*p =* 0.38 for trend, Table 2). Younger age was associated with a reduced likelihood of hospitalisation (HR per year 0.98; 95% CI 0.97 to 0.99, *p* < 0.001), as was alcohol consumption (HR 0.55; 95% CI 0.40 to 0.76, *p* < 0.001) and 5‐aminosalicylate use at baseline (HR 0.59; 95% CI 0.39 to 0.87, *p* = 0.0090). Glucocorticosteroid use at baseline was associated with an increased likelihood of hospitalisation (HR 1.92; 95% CI 1.27 to 2.92, *p* = 0.0020).

When only patients in clinical and biochemical remission or having clinical and biochemical activity at baseline were considered, 38 (21.2%) of 179 patients required hospitalisation during a mean follow‐up of 7.5 years in all patients. Rates of hospitalisation due to uncontrolled IBD activity were highest in those with clinical and biochemical disease activity and symptoms of one (32.0%) or two (38.5%) common mental disorders (Table 3). Following multivariate Cox regression, the likelihood of requiring hospitalisation due to uncontrolled IBD activity was significantly higher only in patients with clinical and biochemical disease activity and symptoms of two common mental disorders at baseline (HR 6.20; 95% CI 1.88 to 20.4, *p* = 0.0030). Sensitivity analyses with a FC of < 250 mcg/g used to define biochemical remission and ≥ 250 mcg/g to define biochemical activity are provided in Table S2.

### 3.4 Intestinal Resection due to Uncontrolled IBD Activity

In total, 90 (12.9%) of 695 patients required intestinal resection due to uncontrolled IBD activity during a mean follow‐up of 7.9 years in all patients, and a median time to intestinal resection of 2.5 years. Rates of intestinal resection were highest in patients with clinical activity and symptoms of one or two common mental disorders (23.9% and 21.7%, respectively, *p =* 0.0020 for trend, Table 2). Following multivariate Cox regression, the likelihood of requiring intestinal resection due to uncontrolled IBD activity was not significantly different between the groups (*p* = 0.14 for trend, Table 2). Glucocorticosteroid use was the only factor associated with an increased likelihood of requiring intestinal resection (HR 2.27; 95% CI 1.28 to 4.01, *p* = 0.0050).

When only patients in clinical and biochemical remission or having clinical and biochemical activity at baseline were considered, 16 (8.8%) of 181 patients required intestinal resection during a mean follow‐up of 8.3 years in all patients. Rates of intestinal resection were highest in patients with disease activity and symptoms of one or two common mental disorders (20.0% and 15.0%, respectively, Table 3). However, after multivariate Cox regression, the likelihood of requiring intestinal resection was not significantly different between the groups, although it remained highest in those with clinical disease activity and symptoms of one or two common mental disorders at baseline (*p =* 0.29 for trend, Table 3). Sensitivity analyses with a FC of < 250 mcg/g used to define biochemical remission and ≥ 250 mcg/g to define biochemical activity are provided in Table S2.

### 3.5 Hospitalisation or Intestinal Resection due to Uncontrolled IBD Activity

In total, 184 (26.6%) of 692 patients were hospitalised or required intestinal resection due to uncontrolled IBD activity during a mean follow‐up of 7.0 years in all patients, with a median time to hospitalisation or intestinal resection of 2.3 years. Patients with clinical disease activity and symptoms of one or two common mental disorders had the highest rates (37.1% and 43.2%, respectively, *p =* 0.0020 for trend, Table 2). After multivariate Cox regression, however, there was no statistically significant difference between the groups, although the rate remained highest in those with clinical disease activity and symptoms of two common mental disorders (HR 1.79; 95% CI 0.99 to 3.23). Younger age (HR per year 0.98; 95% CI 0.97 to 0.99, *p* < 0.001) alcohol consumption (HR 0.55; 95% CI 0.40 to 0.75, *p* < 0.001), and 5‐aminosalicylate use at baseline (HR 0.57; 95% CI 0.39 to 0.85, *p* = 0.0060) were associated with a reduced likelihood of hospitalisation or intestinal resection, and glucocorticosteroid use at baseline (HR 1.99; 95% CI 1.32 to 2.99, *p* < 0.001) an increased likelihood.

**FIGURE 5:**
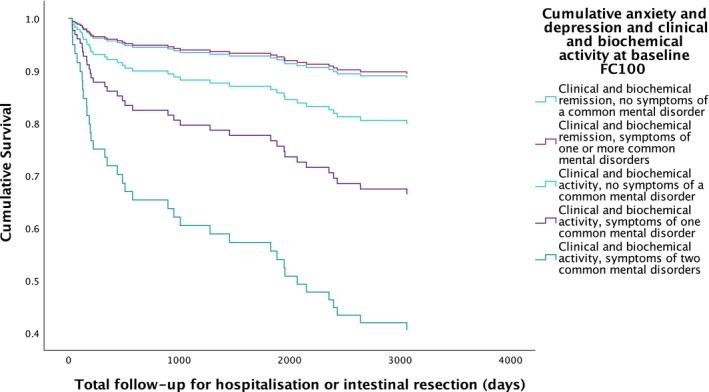
Survival analysis for hospitalisation or intestinal resection due to uncontrolled ibd activity according to combined clinical and biochemical disease activity status and presence or absence of symptoms of a common mental disorder at baseline.

When only patients in clinical and biochemical remission or having clinical and biochemical activity at baseline were considered, 39 (21.7%) of a total of 180 patients were hospitalised or required intestinal resection due to uncontrolled IBD activity during a mean follow‐up of 7.5 years in all patients. Rates were highest in patients with clinical and biochemical disease activity and symptoms of one or two common mental disorders (32.0% and 42.9%, respectively). Following multivariate Cox regression, patients with clinical and biochemical disease activity and symptoms of two common mental disorders were significantly more likely to require hospitalisation or intestinal resection due to uncontrolled IBD activity (HR 7.46; 95% CI 2.41 to 23.2, *p <* 0.001) (Figure 5). Sensitivity analyses with a FC of < 250 mcg/g used to define biochemical remission and ≥ 250 mcg/g to define biochemical activity are provided in Table S2.

### 3.6 All‐Cause Mortality

Of 702 patients, a total of 57 (8.1%) died during a mean longitudinal follow‐up of 8.8 years in all patients, with a median time to death of 5.3 years. Patients with clinical activity and symptoms of two common mental disorders had the highest rates of death (16.7%), but this did not reach statistical significance (*p* = 0.011 for trend, Table 2). However, following multivariate Cox regression, mortality rates were significantly higher in this group of patients (HR 6.97; 95% CI 2.43 to 20.0, *p* < 0.001, Table 2). Increasing age was the only other baseline characteristic associated with an increased risk of death (HR per year 1.12; 95% CI 1.09 to 1.15, *p* < 0.001).

When only patients in clinical and biochemical remission or having clinical and biochemical activity at baseline were considered, 19 (10.4%) of 182 patients died during a mean longitudinal follow‐up of 8.9 years in all patients. Mortality was highest in patients with clinical and biochemical disease activity and symptoms of two common mental disorders (26.7%), but this was not statistically significant (*p* = 0.061 for trend, Table 3). However, after multivariate Cox regression, mortality was significantly higher in patients with clinical and biochemical disease activity and symptoms of either one (HR 33.6; 95% CI 3.83 to 294, *p =* 0.0020, Table 3) or two common mental disorders (HR 57.3; 95% CI 7.58 to 433) (*p* < 0.001, Table 3). Sensitivity analyses with a FC of < 250 mcg/g used to define biochemical remission and ≥ 250 mcg/g to define biochemical activity are provided in Table S2.

## 4 Discussion

This longitudinal follow‐up study has examined the cumulative impact of clinical disease activity, clinical and biochemical activity, and psychological health on adverse disease outcomes in patients with IBD over a mean follow‐up of more than 8 years. Not only were the absolute numbers of almost all the events of interest numerically higher in those with clinical disease activity and symptoms of at least one common mental disorder at baseline, but also increasing psychological burden, as defined by the presence of symptoms of two common mental disorders, was associated with the highest number of adverse disease outcomes in most of our analyses. Even after controlling for all baseline characteristics, individuals with clinical disease activity and symptoms of two common mental disorders were significantly more likely to experience a flare or need for glucocorticosteroid prescription or escalation of medical therapy due to uncontrolled IBD activity and had higher all‐cause mortality. Furthermore, individuals with clinical and biochemical activity and symptoms of two common mental disorders were significantly more likely to experience a flare or need for glucocorticosteroid prescription, escalation of medical therapy, hospitalisation, a composite of hospitalisation or intestinal resection or death. Results were broadly similar when we used a FC of < 250 mcg/g to define biochemical remission and ≥ 250 mcg/g to define biochemical activity, although it should be pointed out that this threshold is debated, and it would also lead to more individuals with potential biochemical activity being assigned to the biochemical remission categories, which may bias the results of the study towards the null hypothesis.

As we are the sole provider of IBD‐related care to all recruited participants, and with access to the involved individuals' electronic medical records, it is likely that we have captured most of the adverse disease outcomes occurring among these patients. Investigators assessing adverse disease outcomes were blinded to participants' baseline questionnaire data to minimise bias when assessing the occurrence of the endpoints of interest during longitudinal follow‐up. Performing multivariate Cox regression analysis to control for all baseline demographic and IBD‐related data, as well as somatoform symptom‐reporting, means our findings are unlikely to be confounded by these other potential influencers of adverse disease outcomes. Even though our unit conforms to recommendations to seek definitive evidence of disease activity prior to any escalation of medical therapy [36], flare of disease activity and, to a lesser extent, escalation of medical therapy are more contingent on patient‐reported outcome measures, which introduce a degree of subjectivity and could be influenced by patient over‐reporting [28, 37]. Definitions of what constitutes a flare may also vary between centres. The sample size and the extended follow‐up period of the study mean that the chance of rarer, but more objective, events of interest occurring during follow‐up was maximised. Finally, various cut‐offs are applied in clinical practice to determine biochemical disease activity, but our use of a FC of < 100 mcg/g of stool to define biochemical remission has a greater sensitivity than higher cut‐offs. This means the likelihood of overestimating the number of patients in remission at baseline is much less likely, and the outcomes observed are more likely to be genuinely due to the combined effects of disease activity and adverse psychological health [38]. The cumulative impact of mood and disease activity on the more objective disease outcomes, including hospitalisation or intestinal resection and death, identified in these analyses, therefore underscores the importance of brain‐gut effects in IBD.

Conducting the study alongside routine clinical care introduces some weaknesses. Despite every effort to eliminate bias, existing documentation could not be redacted from participant's medical records prior to review. It is, therefore, possible that assessors may have seen evidence of an existing diagnosis of a common mental disorder, which could have influenced interpretation of more subjective endpoints. Similarly, participants were managed by several different physicians throughout the study, which introduces a degree of interobserver variation. For instance, there are likely to be differing thresholds assigned to the more subjective endpoints of interest in clinical practice. Participants' medical records were reviewed by two different assessors during longitudinal follow‐up, which could also introduce a degree of interobserver variation, although pre‐defined criteria for each of the adverse disease outcomes were adhered to by both assessors. Inferring a diagnosis of a common mental disorder using the HADS questionnaire responses from a single point in time, rather than performing a clinical assessment to establish a formal diagnosis, could be criticised for being overly simplistic. However, this would have been infeasible given the sample size. In addition, in a recent study examining trajectories of HADS anxiety or depression scores in patients with IBD, only 10% of patients with abnormal scores at baseline had an improvement during longitudinal follow‐up [20]. This suggests a single assessment may be stable in the majority of patients with IBD. Only half of patients provided baseline FC samples, and, because of our stringent definition of clinical and biochemical remission or activity, these analyses may be inadequately powered or give imprecise results, as reflected by the wide 95% CIs. Therefore, some of the results, particularly those for all‐cause mortality, should be interpreted with caution. We did not collect data on other drugs that may influence the activity or prognosis of IBD, such as opioids or non‐steroidal anti‐inflammatory drugs [39]. Finally, repeat FC measurements were not performed during longitudinal follow‐up to assess changes in disease activity. However, this was infeasible with an average follow‐up of 8.1 years, and the fluctuating course of IBD is both unlikely to have been a reliable determinant of overall disease activity and to add to the study considering the objective adverse disease outcomes we examined.

These results confirm our findings from two of our previous studies but add to them. First, we have demonstrated previously that adverse psychological health at baseline influences the natural history of IBD negatively, and to a similar degree to inflammatory burden [27]. Second, we have also shown that, among patients with IBD in biochemical remission, there is a cumulative effect of psychological co‐morbidity at baseline on the likelihood of adverse disease outcomes [14]. The current study examines the impact of both the presence and the degree of psychological co‐morbidity at baseline on these endpoints according to both clinical and biochemical disease activity. Compared with existing studies examining brain‐gut and gut‐brain effects in IBD [19], this study has one of the longest periods of follow‐up and utilised markers of biochemical remission to determine baseline disease activity alongside traditional clinical disease activity indices. The existing body of research examining brain‐gut effects in IBD demonstrates an association between symptoms of a common mental disorder and future adverse disease outcomes [17, 40, 41, 42]. However, most prior studies have delineated patients only by measures of psychological health [19], and have not assessed whether there is a cumulative impact of disease activity, which is also a driver of morbidity and is, in itself, associated with a higher prevalence of symptoms of a common mental disorder [25].

The sole use of clinical disease activity indices to determine underlying inflammatory activity is another inherent flaw of many existing studies examining brain–gut effects in IBD [19]. These correlate poorly with underlying mucosal inflammation and are now considered to constitute low value care when used in isolation [28, 36, 37]. An exception to this is a study by Mules et al. [43], the results of which are in contrast to those from our study. The authors demonstrated that symptoms of psychological illness at baseline were associated with an increased symptom burden in IBD, but not with endoscopic or biochemical disease activity, or the development of subsequent disease activity, as determined by a repeat FC at 6 months. However, like many other studies in the field [19], the duration of follow‐up was short and the number of included patients relatively small. Other groups have reported that severity in IBD can be characterised by other factors beyond inflammation [44], incorporating baseline medications and some blood results, and this can be used to predict prognosis [45, 46, 47]. Finally, most studies examining brain –gut or gut –brain interactions are performed in established cohorts of patients with IBD [19]. This means that patients may have already experienced an adverse disease course, which could have impacted their psychological health. Any future association between psychological health and prognosis may, therefore, arise due to confounding. Inception cohorts that assess psychological health at the time of a diagnosis of IBD will be required to disentangle this complex relationship.

The importance of inflammatory burden in IBD is undisputed. However, the observation that psychological health appears not only to affect disease outcomes to a similar magnitude to disease activity but also has a cumulative effect alongside disease activity challenges the absence of psychological health as a treatment target [3]. Given the consistent association between disease activity and poor psychological health in IBD, considering both the inflammatory and psychological burden of the disease is key, particularly as treatment of the two may differ considerably. Brain‐gut behavioural therapies are effective in irritable bowel syndrome [48, 49]. They may have optimal efficacy in IBD when delivered to selected patients with evidence of pre‐existing psychological co‐morbidity [50], which is of particular relevance to healthcare systems with finite resources, as the blanket use of psychological therapies is probably unrealistic. Our results suggest that the impact of adverse psychological health is greatest in patients with disease activity, yet only four randomised controlled trials (RCTs) have evaluated the role of such therapies in the context of active IBD, and none recruited patients with evidence of psychological co‐morbidity [51, 52, 53, 54]. To integrate a biopsychosocial model of care in routine IBD practice successfully requires further assessment of these treatments in this particular group of patients. Similarly, neuromodulators have a good evidence base in disorders of gut‐brain interaction [55, 56, 57, 58]. However, there is a lack of RCTs in IBD, and although they appear to demonstrate positive effects in treating psychological and somatic symptoms, limited conclusions can be made regarding their influence on the disease course [59]. This should be a priority area for future IBD research, particularly given the favourable safety profile seen in similar RCTs conducted in irritable bowel syndrome [55].

In summary, our study demonstrates that, even when stringent and objective assessments are applied to determine underlying disease activity, genuine brain –gut effects are apparent in IBD. Not only does adverse psychological health appear to influence the natural history of the disease to a similar magnitude to inflammatory activity, but also this is cumulative, with patients with symptoms of more than one common mental disorder at the greatest risk of future adverse disease outcomes. That most of these endpoints were not significantly higher in patients with active disease alone highlights alternative therapeutic targets in IBD, which do not feature in current guidelines but, if utilised correctly, have the potential to improve outcomes for some patients. Given the prevalence of common mental disorders among patients with IBD, our findings suggest that focusing solely on the inflammatory aspects of UC or CD will result in unmet needs among a substantial proportion of patients and may contribute to adverse disease outcomes. There are ever‐expanding options to identify and treat persistent mucosal inflammation, but strategies to identify and manage co‐existing poor psychological health are also needed if we are to provide holistic care for patients with IBD.

## Author Contributions

**Christy Riggott:** writing – original draft, data curation, formal analysis. **Keeley M. Fairbrass:** data curation. **David J. Gracie:** conceptualization, investigation, funding acquisition, methodology, writing – review and editing, supervision. **Alexander C. Ford:** supervision, formal analysis, writing – review and editing, conceptualization, funding acquisition, investigation.

## Ethics Statement

This study involves human participants and was approved by the Yorkshire and Humber ethics committee reference 12/YH/0443/AM03.

## Conflicts of Interest

The authors declare no conflicts of interest.

## Supporting information


Table S1.

## Data Availability

The data that support the findings of this study are available on request from the corresponding author. The data are not publicly available due to privacy or ethical restrictions.
