# Supplementary material for: Cumulative Impact of Clinical Disease Activity, Biochemical Activity and Psychological Health on the Natural History of Inflammatory Bowel Disease During 8 Years of Longitudinal Follow‐Up
Source: Aliment Pharmacol Ther. 2025 Mar 9;61(10):1635–48. doi: 10.1111/apt.70068 (PMC12013785; doi:10.1111/apt.70068)
Supplement: Supplementary file 1 — Table S1. [file APT-61-1635-s001.docx]

**Supplementary Table 1. Baseline Patient Characteristics According to Both Combined Clinical and Biochemical Disease Activity and Symptoms of a Common Mental Disorder at Baseline.**

|  | **Combined Clinical and Biochemical Remission** | | **Combined Clinical and Biochemical Activity** | | |  |
| --- | --- | --- | --- | --- | --- | --- |
|  | **No symptoms of a common mental disorder**  **(n=83)** | **Symptoms of a common mental disorder**  **(n=16)** | **No symptoms of a common mental disorder**  **(n=46)** | **Symptoms of a common mental disorder**  **(n=27)** | **Symptoms of two common mental disorders**  **(n=15)** | ***P* value*** |
| **Mean age (SD)** | 50.9 (18.0) | 45.8 (14.1) | 51.8 (15.4) | 45.4 (14.2) | 50.2 (18.2) | 0.44 |
| **Female sex (%)** | 39 (47.0) | 10 (62.5) | 26 (56.5) | 21 (77.8) | 8 (53.3) | 0.084 |
| **Married or cohabiting (%)** | 54 (65.1) | 12 (75.0) | 33 (71.7) | 20 (74.1) | 8 (53.3) | 0.57 |
| **University graduate/ professional (%)** | 28 (33.7) | 5 (31.3) | 13 (28.9) | 8 (29.6) | 4 (26.7) | 0.97 |
| **Tobacco user (%)** | 8 (9.6) | 1 (6.3) | 2 (4.3) | 8 (29.6) | 4 (26.7) | 0.0070 |
| **Alcohol user (%)** | 54 (65.1) | 14 (87.5) | 32 (71.1) | 16 (59.3) | 5 (33.3) | 0.023 |
| **CD (%)** | 42 (50.6) | 11 (68.8) | 21 (45.7) | 13 (48.1) | 9 (60.0) | 0.54 |
| **CD location (%)**  Ileal (%)  Colonic (%)  Ileocolonic (%) | 6/42 (14.3)  18/42 (42.9)  18/42 (42.9) | 2/11 (18.2)  7/11 (63.6)  2/11 (18.2) | 5/21 (23.8)  5/21 (23.8)  11/21 (52.4) | 7/13 (53.8)  1/13 (7.7)  5/13 (38.5) | 2/9 (22.2)  2/9 (22.2)  5/9 (55.6) | 0.033 |
| **Non-stricturing, non-penetrating CD (%)** | 40/42 (95.2) | 9/11 (81.8) | 17/21 (81.0) | 8/13 (61.5) | 7/9 (77.8) | 0.015 |
| **Perianal disease (%)** | 2/42 (4.8) | 0/11 (0.0) | 3/21 (14.3) | 0/13 (0.0) | 1/9 (11.1) | 0.35 |
| **UC extent (%)**  Proctitis  Left-sided  Extensive | 9/41 (22.0)  19/41 (46.3)  13/41 (31.7) | 2/5 (40.0)  3/5 (60.0)  0/5 (0.0) | 5/25 (20.0)  11/25 (44.0)  9/25 (36.0) | 2/14 (14.3)  7/14 (50.0)  5/14 (35.7) | 1/6 (16.7)  4/6 (66.7)  1/6 (16.7) | 0.84 |
| **5-aminosalicylate use (%)** | 44 (53.0) | 8 (50.0) | 24 (52.2) | 12 (44.4) | 6 (40.0) | 0.86 |
| **Immunomodulator use (%)** | 31 (37.3) | 5 (31.3) | 16 (34.8) | 9 (33.3) | 3 (20.0) | 0.78 |
| **Biologic use (%)** | 13 (15.7) | 3 (18.8) | 10 (21.7) | 6 (22.2) | 1 (6.7) | 0.66 |
| **Glucocorticosteroid use (%)** | 2 (2.4) | 1 (6.3) | 8 (17.4) | 2 (7.4) | 1 (6.7) | 0.047 |
| **High levels of somatoform symptom-reporting on PHQ-15 (%)** | 5 (6.0) | 3 (20.0) | 5 (11.9) | 13 (50.0) | 7 (50.0) | <0.001 |

*One-way analysis of variance for comparison of continuous data, χ^2^ for comparison of categorical data across all four groups.

**Supplementary Table 2. Adverse Disease Outcomes in Patients According to Combined Clinical and Biochemical Disease Activity Status, using an FC<250mcg/g or ≥250mcg/g, and Presence or Absence of Symptoms of a Common Mental Disorder at Baseline.**

|  | **Combined clinical and biochemical remission** | | **Combined clinical and biochemical activity** | | |  |
| --- | --- | --- | --- | --- | --- | --- |
|  | **No symptoms of a common mental disorder** | **Symptoms of a common mental disorder** | **No symptoms of a common mental disorder** | **Symptoms of a common mental disorder** | **Symptoms of two common mental disorders** | ***P* value** |
| **Flare of disease activity or glucorticosteroid prescription (%)** | 48/109 (44.0) | 18/29 (62.1) | 11/15 (73.3) | 9/10 (90.0) | 3/4 (75.0) | 0.010* |
| **Multivariate HR for flare of disease activity or glucorticosteroid prescription (95% CI)** | 1.00 (reference) | 1.82 (0.98-3.40) | 3.61 (1.66-7.81)† | 6.33 (2.31-17.3)‡ | 3.87 (1.05-14.3) | <0.001 |
| **Escalation of medical therapy due to uncontrolled IBD activity (%)** | 49/109 (45.0) | 18/29 (62.1) | 18/22 (81.8) | 8/10 (80.0) | 5/6 (83.3) | 0.0030* |
| **Multivariate HR for escalation of medical therapy due to uncontrolled IBD activity (95% CI)** | 1.00 (reference) | 1.79 (0.98-3.26) | 3.87 (2.07-7.23)‡ | 2.48 (0.94-6.52) | 5.07 (1.83-14.1)† | <0.001 |
| **Hospitalisation due to uncontrolled IBD activity (%)** | 17/109 (15.6) | 6/29 (20.7) | 9/37 (24.3) | 6/14 (42.9) | 2/9 (22.2) | 0.18* |
| **Multivariate HR for hospitalisation due to uncontrolled IBD activity (95% CI)** | 1.00 (reference) | 1.44 (0.53-3.93) | 1.39 (0.57-3.38) | 5.67 (1.85-17.4)† | 3.23 (0.65-16.1) | 0.042 |
| **Intestinal resection due to uncontrolled IBD activity (%)** | 4/109 (3.7) | 2/29 (6.9) | 4/37 (10.8) | 4/14 (28.6) | 3/11 (27.3) | 0.0030* |
| **Multivariate HR for intestinal resection due to uncontrolled IBD activity (95% CI)** | 1.00 (reference) | 1.53 (0.25-9.38) | 2.46 (0.52-11.5) | 13.2 (2.55-67.9)† | 15.31 (2.42-96.8)† | 0.0090 |
| **Hospitalisation or intestinal resection (%)** | 17/109 (15.6) | 6/29 (20.7) | 9/37 (24.3) | 6/14 (42.9) | 3/10 (30.0) | 0.15* |
| **Multivariate HR for hospitalisation or intestinal resection (95% CI)** | 1.00 (reference) | 1.46 (0.54-3.98) | 1.42 (0.58-3.45) | 5.89 (1.94-17.8)† | 5.05 (1.27-20.1) | 0.015 |
| **Death (%)** | 16/111 (14.4) | 1/29 (3.4) | 1/37 (2.7) | 2/14 (14.3) | 4/11 (36.4) | 0.017* |
| **Multivariate HR for death (95% CI)** | 1.00 (reference) | 0.79 (0.09-6.73) | 0.35 (0.05-2.79) | 7.25 (1.30-40.3) | 10.3 (2.27-46.5)† | 0.0070 |

*For comparison across all four groups.

†*P*<0.01 versus reference category.

‡*P*<0.001 versus reference category.
